# Integrating behavioural science and design thinking to develop a water treatment innovation in Malawi healthcare: a case study of the intervention design process

**DOI:** 10.1080/16549716.2026.2726126

**Published:** 2026-09-09

**Authors:** Chiara Pittalis, Christabel Kambala, Elizabeth Kogoya, Desire C. Mussa, Antonio Jaén Osuna, Kevin G. McGuigan, Jakub Gajewski

**Affiliations:** a School of Population Health, RCSI University of Medicine and Health Sciences, Dublin, Ireland; b Public and Environmental Health Sciences Department, School of Science and Technology, Malawi University of Business and Applied Sciences, Blantyre, Malawi; c Department of Physiology and Medical Physics, RCSI University of Medicine and Health Sciences, Dublin, Ireland; d Centre for Global Surgery, University of Stellenbosch, Cape Town, South Africa

**Keywords:** Clean water, maternal health, participatory research, SODIS, Africa

## Abstract

Access to safe water is essential for maternal and neonatal care, yet many healthcare facilities (HCFs) in low- and middle-income countries (LMICs) face severe water scarcity, undermining service delivery and patient outcomes. Malawi exemplifies this challenge. While technologies such as solar disinfection of harvested rainwater (HRW-SODIS) offer potential solutions, mere deployment does not guarantee adoption or sustainability. This study reports on experiences and lessons learned from the design of the SURG-Water project, which is testing, for the first time, the application of HRW-SODIS in clinical settings. Rather than focusing solely on technical performance, the project employed a co-design process grounded in behavioural science and design thinking to align both the technology and its deployment strategy with facility workflows, user preferences and socio-cultural norms. Two maternal HCFs were purposively selected for the pilot, and a participatory design process involving health workers, community leaders and government stakeholders guided iterative HRW-SODIS system development. Site assessments, interviews, and focus groups revealed behavioural, infrastructural, and institutional factors that shaped design choices. This process produced not just a water treatment solution, but an integrated strategy including staff training, educational materials, and community sensitisation. The work demonstrates a clear pathway from scientific concept to contextually grounded intervention design. The SURG-Water model offers a replicable example of how interdisciplinary, user-centred methods can be used to bridge knowledge and practice in resource-constrained healthcare settings. While SODIS has existed for years, it remains underused; this approach provides a practical step toward addressing real-world barriers to uptake, starting from its design.

## Paper Context

- **Main findings:** This is the first study ever on rainwater solar disinfection in clinical settings, documenting needs and potential for improving safe water access in rural health facilities.
- **Added knowledge:** The study advanced a co-design methodology that directly linked research findings with facility-level implementation planning, producing a replicable model for the introduction of new technologies in low-resource clinical environments.
- **Global health impact for policy and action:** The study applied behavioural and systems insights to develop an evidence-based deployment strategy, identifying key factors for user acceptance, ownership, and sustained use yielding practical lessons for global practitioners and decision-makers to support adoption and roll-out of solar disinfection technologies.

## Background

Access to safe, reliable water and adequate sanitation and hygiene (WASH) are fundamental for health [1], and critical for the provision of maternal and neonatal care. Every year, over 16 million women in the least-developed countries give birth in healthcare facilities (HCFs) without adequate water and hygiene measures, putting their lives and their babies at risk of preventable infections [1]. An estimated 287,000 maternal deaths occurred globally in 2020 [2]. Although the causes of maternal mortality are diverse, previous estimates suggest that infections, predominantly sepsis, account for approximately 11% of maternal deaths [3]. Infections are also responsible for about 30% of neonatal deaths, with lack of access to water a major contributor [1]. These figures highlight the importance of clean healthcare facilities to prevent infections and improve health outcomes.

The escalating Climate Crisis is worsening the already severe water supply challenges facing HCFs across the globe, particularly in resource-limited settings [1]. Extreme weather patterns – such as droughts, erratic rainfall and flooding – are making access to water more unpredictable. Water sources are either drying up, becoming increasingly polluted, or are being depleted by water-intensive industries, exacerbating the crisis [4]. This creates an urgent need for innovative strategies to safeguard and improve access to clean water, especially in vulnerable healthcare settings.

In sub-Saharan Africa (SSA) the situation is dire – more than 1 in 4 HCFs have limited or no access to water services [1], and a study in East Africa found that fewer than 30% of delivery rooms had access to water [5]. Sharp inequalities between urban and rural areas and across care levels also exist. Primary HCFs (e.g. health centres and rural hospitals), which serve as the first point of care for rural populations and are critical to maternal and newborn health, face considerably lower WASH coverage than higher-level hospitals [1].

In the current unstable and changing climate, HCFs need adaptive management strategies to be resilient and to protect the health of the communities they serve [4]. Yet, this is not an easy task in resource-limited settings. For example, Malawi, one of the world’s poorest countries, struggles with a heavy disease burden, high vulnerability to extreme weather and severe land degradation – all of which are expected to worsen due to the Climate Crisis [6–8]. These challenges risk further exacerbating the already large gaps in water supply at HCFs [9]. No comprehensive data exist on the effects of poor water access at HCFs, highlighting a significant evidence gap and the urgent need for innovative solutions. Despite increasing research in the WASH sector, there is also limited evidence on interventions specifically targeting HCFs [10].

The SURG-Water Project is attempting to respond to these challenges through the design of a technology to solar disinfect harvested rainwater using solar UV as a ‘renewable’ energy source. The proposed solution centres on the development and demonstration of the potential of a large volume (>200 L) transparent batch solar water disinfection (SODIS) reactor, deployed to treat harvested rainwater (HRW) collected on-site in HCFs in Malawi. It was piloted in two sites in Southern Malawi in order to be tailored to the needs of district HCFs within the rural context. The goal is to provide a backup clean water system so that health facilities can continue to function despite extreme weather events or interruptions to mains water supply, ensuring the availability of safe water for essential maternal health services.

### A human-centred approach

The successful introduction of new technologies, such as solar disinfected harvested rainwater (HRW-SODIS) systems, demands careful, context-sensitive planning and a deep understanding of the local context [11,12]. Meaningful engagement with stakeholders through multi-disciplinary, participatory research is essential [12] - not only to understand how the technology can be used but also to identify the various factors that may influence its subsequent adoption and uptake [11]. Integrating design thinking [13,14] and behavioural science [15] presents an opportunity for methodological advancement in WASH research, to ensure that new technologies are both effective and embraced by the intended users.

Both design thinking and behavioural science emphasise the importance of a human-centred approach [16] and recognises the roles of attitudes and motivation, among other things, in the behaviour of target users towards an innovation [15,17,18]. They differ, however, in approaches. Design thinking is iterative and collaborative, and focuses on understanding users’ needs, mapping their experiences and actively prototyping solutions with them through ongoing feedback [19]. Despite its growing application across disciplines as a methodology well suited to encourage innovation [20], the specific mechanisms through which it might improve innovation outcomes [21] and support sustainability [22] have not received significant attention.

In contrast, behavioural science is more linear. It uses theory and evidence to design interventions to achieve specific outcomes, like technology adoption, through behaviour change, with less emphasis on ongoing feedback [16,19]. While nudging a behaviour change may indeed be necessary when introducing an innovation, relying too heavily on it may overlook the essential pre-development work needed to ensure the innovation is relevant and fits the local cultural context [12].

Given their relative strengths and weaknesses, together these approaches have the potential to create a powerful synergy. Design thinking helps to build a deeper understanding of users’ needs and the local context, ensuring solutions are relevant and desirable, while behavioural science sheds light on the psychological and social factors that influence adoption and sustained use [16]. By addressing attitudes, motivations and barriers, these approaches can increase the likelihood that the technology will be effectively adopted and seamlessly integrate into users’ lives.

The aim of this paper is to share our experiences and key lessons learned from integrating design thinking and behavioural science in the development of a SODIS-based harvested rainwater treatment system for rural healthcare facilities in Malawi. By combining these interdisciplinary approaches, we provide key insights into designing technology that meets the unique needs of underserved communities while overcoming adoption barriers. The goal is to advance broader scientific discourse on methodological strategies for introducing innovations in resource-limited settings to maximise potential impact.

## Methods

Our case study followed an iterative participatory design process, integrating principles of design thinking [13,14] with behavioural science [15] to address water shortages in district-level HCFs. The specific objectives were:

1. To assess current water challenges in Southern Malawi, focusing on water sources (availability, reliability and water quality) for maternal care across a sample of HCFs.
2. To document the impact of water scarcity on maternal health services.
3. To identify factors that may influence the introduction and adoption of the proposed rainwater treatment technology.

This was necessary for shaping the solution concept, gathering baseline data for its future evaluation, and providing valuable lessons to refine the proposed intervention. The research process was organised into three phases, aligned with the five core principles of design thinking [23] as outlined in Table 1.

**Table 1.** Overview of the research process.

Phase 1: Problem Understanding and Contextualisation	Phase 2: Ideation and Intervention Design		Phase 3: Prototyping and Testing	
(i) **EMPATHISE** with users to understand their context, behaviours, experiences, and needs. This initial research aimed to establish a clear baseline, distinguishing between users’ expectations and true needs, while identifying what was feasible within the given timeline and resources.	(ii) Analyse and process the collected information to **DEFINE** the core problem and specify/narrow down the research question.	(iii) Collaborate with stakeholders to **GENERATE IDEAS** on how best to address the identified challenge. This was achieved through active group work and facilitated discussions bringing together a variety of perspectives.	(iv) Create a **PROTOTYPE** of the proposed solution to test the technology in practice.	(v) **TEST** and evaluate the prototype with users, gather feedback, and iteratively refine the solution based on insights to improve the design.
Data collection: site inspections, observations, interviews with health facility staff and focus groups with patients		Data collection: workshop and meetings with relevant stakeholders		Data collection: water quality analysis, site inspections, interviews with health facility staff and stakeholders’ workshops
Variables of interest: Water access Water usage/needs Water quality Impact of current water supply on care delivery and patients’ experiences		Variables of interest: barriers, facilitators and opportunities for introduction of the technology in the testing sites		Variables of interest: Fidelity Feasibility Acceptability Adoption Effectiveness (technical and non-technical) Areas for improvement
Participants: Inspections: average of 3 facility representatives at each site Interviews: 3 staff members (all females) from Chimvu HC and 4 (1 male, 3 females) from Thyolo DH Focus groups: 9 female patients in Chimvu HC and 24 in Thyolo DH		Participants in stakeholders’ consultations: 36 (21 male, 15 female)		

This paper reports on the first two phases of the research process; the third element, the testing, is discussed in a separate publication (forthcoming).

### Study settings

The Southern region of Malawi was chosen as the study setting, considering the relatively high vulnerability of the local population to climate-related hazards [24]. Three districts were selected for the baseline situation assessment based on their geographical characteristics (sun exposure, rainfall, distance from Blantyre), and difficulties with water supply in HCFs reported in another study by our team [25]: Mulanje district, Thyolo district and Chikwawa district. Characteristics are summarised in Table 2.

**Table 2.** Key characteristics of the sampled districts.

District	Population size	% of population living in rural areas	No. of HCFs
Mulanje district	684,107	97.8%	16
Thyolo district	721,456	97.2%	14
Chikwawa district	564,684	97.7%	18

Note: Data from the 2018 Malawi Population and Housing Census [26].

### Data collection

The research was conducted by a multidisciplinary team comprising local and international experts in HRW-SODIS, microbiology, engineering, social science and maternal health. Data collection involved multiple methods, as follows.

#### Site inspections

We followed a stepwise approach, starting with site inspections of the district hospitals (DHs) in each district (*n* = 3) as these were the key referral points for lower-level peripheral facilities. Through snowballing, we then selected other three nearby health centres (HCs) and rural hospitals (RH) with reported intermittent or absent access to water. Facilities with a connection to a reliable and uninterrupted municipal treated water supply were excluded. DH and HCF inspections were carried out from February to May 2023.

The site inspections focused on the operating theatre and labour wards at the district hospitals, and on pre- and post-natal wards and delivery rooms at the lower-level facilities (which are smaller in size). We consulted with a range of staff such as WASH coordinators, deputy WASH coordinators, environmental Officers, facility in-charge, maternity in-charge/nurses, surgical providers (at the hospitals) and estates officers. Thyolo district was the district chosen to deliver the intervention based on the proceedings of the site inspections, and further data collection was conducted in this district.

#### Interviews and focus groups

The site inspections were followed by interviews and focus group discussions (FGDs) with health facility staff and patients to collect further data on the lived experience of participants. These were conducted in March 2023. To capture a broad range of experiences, we selected one district hospital (Thyolo DH) and one health centre (Chimvu HC), ensuring that we could examine the differences in conditions and challenges faced across different facility types. The interviews involved 7 health providers that deliver maternity care, i.e. maternity unit in-charge, hospital attendants, nurse midwives, a surgical provider and theatre nurses; selected with the support of the District Health Officer. The FGDs included 33 female patients, recruited through gatekeepers at the health facilities. They were all expectant mothers, with the exception of one patient who had delivered the night before. Pregnant women who were ill or in any form of discomfort were excluded.

Topics explored with patients covered the availability and quality of water since they had arrived at the HCF, as well as whether these issues had affected them and their baby and if so, how. Topics explored with health providers included discussions over availability of water infrastructure at the HCF, any issues with water quality, and how these impacted their ability to provide care for their patients. Suggestions were also gathered on the best use of proposed technology to meet their needs.

Staff interviews were conducted in English, while FGDs with patients were conducted in the local language (Chichewa), with simultaneous English translation since not all participants spoke English. This was facilitated by the SURG-Water Malawi Lead and the Project Coordinator who are bilingual in Chichewa and English. The researchers involved in FGDs were all females, which reduced gender and social desirability bias. Both interviews and FGDs were conducted in a space away from the nurses and other staff within the facility (either a consultation room or the antenatal ward).

It was known within the clinic that we were researchers; therefore, we opted not to use a recorder when conducting interviews and FGDs to encourage greater rapport and openness given that these were rural and culturally conservative communities, and discussions were conducted within maternity care settings, where topics related to pregnancy, childbirth, hygiene, and personal experiences can be sensitive. While this approach has limitations in terms of verbatim data capture, the decision is consistent with existing research that recognises non-recorded data collection, combined with detailed contemporaneous note-taking, as an appropriate approach in contexts where recording devices may inhibit participant disclosure [27]. Relevant information was written down during the conversation by one of the researchers present in the room. Immediately after the end of the interview/FGD, the notes were cross-checked by the second researcher and modified/integrated as needed.

The sample size was not predetermined as the main goal was to reach information power such that the research questions would be answered.

#### Stakeholder consultations

Consultations with stakeholder groups, including local authorities, health facility managers and staff, traditional leaders, community leaders, representatives from local businesses, NGOs working in the sector, and the Water Board for the Southern Region, were conducted during a central workshop and follow-up meetings in May 2023. The stakeholder list (*n* = 36) was developed in collaboration with, and approved by, the District Health Officer. A topic guide was created to steer the discussions, focusing on exploring local and broader contextual factors that could impact the intervention, as well as jointly identifying key needs and priorities.

### Data analysis

The assessment of the current water situation, maternal needs, and potential for technology installation was based on eight criteria: water access challenges; roof suitability for rainwater collection; sufficient space on facility grounds to accommodate at minimum one 10,000 L harvested rainwater tank, a HRW-SODIS reactor positioned in full, uninterrupted sunlight, and a nearby treated-water holding tank; site security; ease of access to the facility; and potential impact. These criteria were developed by the research team’s prior experience and aligned with WHO guidelines on essential environmental health standards in health care [28]. Each criterion was scored on a scale from 0 (least suitable) to 5 (most suitable), with total scores calculated in MS Excel to provide an overall assessment of each facility – see Supplementary Material for details.

Qualitative data from interviews, FGDs and consultations were analysed in NVivo v12 using reflexive thematic analysis as described in Braun and Clarke [29], supported by a hybrid deductive-inductive coding strategy. Deductive codes were informed by domains derived from the design thinking and behavioural science frameworks underpinning the study, while inductive coding enabled identifications of themes not anticipated by the theoretical framework. These included the local operational context (e.g. availability, reliability and quality of water sources), implications for care delivery, and the impact on both staff and patients, as well as the broader operational context. Within this framework, we applied key principles from the COM-B model to identify factors influencing the desired Behaviour – specifically, the use of treated rainwater by health staff at the participating facilities. This approach emphasises Capability, Opportunity and Motivation as key determinants of behaviour change [15].

### Ethics

This study received ethical approval from the National Committee on Research in the Social Sciences and Humanities in Malawi (approval no. P.03/23/747) and RCSI Research Ethics Committee in Ireland (approval no. REC202301009), and was conducted in accordance with the principles of the Declaration of Helsinki [30]. All participants provided written informed consent prior to participation.

## Results

### Problem understanding and contextualisation

The design thinking process started with a situation assessment to investigate the target population’s challenges and needs. This helped define the problem of access to water from the users’ perspective. In doing so, we also captured any aspects of users’ behaviour that might influence their openness to use (treated) rainwater.

### Local operational context

#### Piped water supply

Results of the site inspections (details in Supplementary Material) showed that all inspected facilities had difficulties in access to water, with the rural and/or remote ones most affected.

The hospitals reported intermittent piped water supply. At Thyolo DH, for instance, interviewed staff explained that the water system linked to an external borehole was non-functional due to broken pipes. The maternity unit’s infrastructure, including 16 toilets, sinks and showers, was inoperable, leaving patients and staff without basic sanitation. This also disrupted essential equipment, such as the sterilising autoclave. Although the operating theatre had a procedure sink connected to an outside tank, this arrangement only provided water for handwashing, leaving the rest of the unit without sufficient water for cleaning and hygiene.

**Figure 1.**
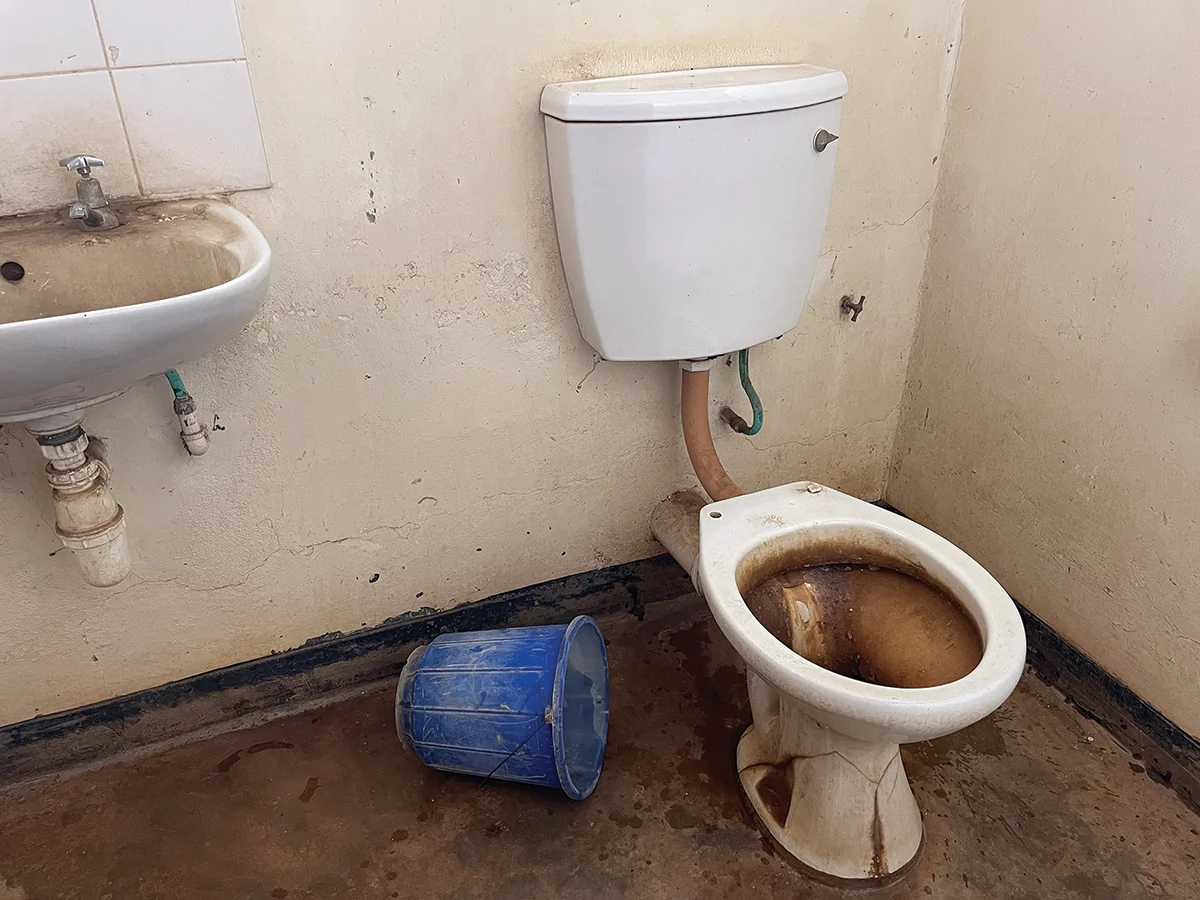
Non-functional toilet facility in a maternity unit resulting from inadequate water supply. The image illustrates how insufficient water availability within the health facility renders sanitation infrastructure unusable and unhygienic, adversely affecting the daily environment of patients and healthcare workers.

Lower-level health facilities, particularly remote ones, had no connection to municipal piped water infrastructure managed by the Water Board (see Figure 1). For instance, Chimvu HC staff reported being without running water for several years due to a broken on-site borehole, which had been out of service since 2020 after repeated breakdowns and pump thefts. Like Thyolo DH, Chimvu HC had non-functional toilets, sinks and no on-site water for the autoclaves.

#### Other water sources

Site inspections and interviews highlighted that most facilities addressed infrastructure deficiencies through various strategies. For example, Thyolo DH relied on a backup borehole on-site, with staff manually fetching water as needed. Chimvu HC, meanwhile, depended largely on patients’ guardians bringing water from a nearby, off-site community borehole, though this source was not exclusive to health staff and patients. This practice created a cycle where each patient discharged was responsible for fetching water for the next.

Similar issues and coping mechanisms, to varying degrees, were observed at the other inspected facilities (Chikwawa DH, Mulanje DH, Thekerani RH, Bvumbwe HC). Despite these mitigation efforts, all inspected facilities reported insufficient water to meet their needs. Hospitals faced challenges with the higher demands of larger patient numbers and staff, as well as specific needs like infection prevention for surgeries (e.g. caesarean sections). To put things in perspective, Thyolo DH staff highlighted that their maternity unit worked day and night and some months they could reach 700 deliveries. On average, 15 operations per day were performed in the operating theatre, between C/S and other maternal surgeries.

On the other hand, health centres relying on community boreholes struggled with limited access, distant location and long-waiting times to fetch water. Although patient numbers were lower than in hospitals, facilities like Chimvu HC also had to meet the needs of staff, many of whom lived on-site due to the facility’s remote location. The reliance on off-site boreholes further compounded challenges, as it meant limited control over water quality. Staff at Chimvu HC, for example, expressed concerns about contamination, as the community borehole was situated near pit latrines, with risks heightened during the rainy season. These concerns grew during a Cholera outbreak in early 2023, particularly after a Cholera screening camp was set up near the borehole by an NGO.

During the site inspections, we observed that rainwater was not utilised at the visited facilities. Stakeholders attributed this to a lack of physical opportunity, such as the absence or dysfunction of rainwater collection tanks and shortages of buckets. Additionally, safety concerns and cultural beliefs within the local communities were identified as factors influencing motivation to use rainwater. These included the perception that rainwater was ineffective for cleaning, unpleasant to use and potentially harmful.

### Implications

The lack of water within the maternity units had far-reaching implications, described below.

#### Burden on staff

As reported by interviewees, lack of water increased workload and time pressure for staff. In Thyolo DH staff spent time fetching water instead of focusing on patient care. Similarly, in Chimvu HC, staff explained that when patients’ guardians were unavailable to help, they had to fetch water themselves from the community borehole. On some occasions, staff were left with minimal water for up to two days because of this.

#### Burden on patient guardians

**Figure 2.**
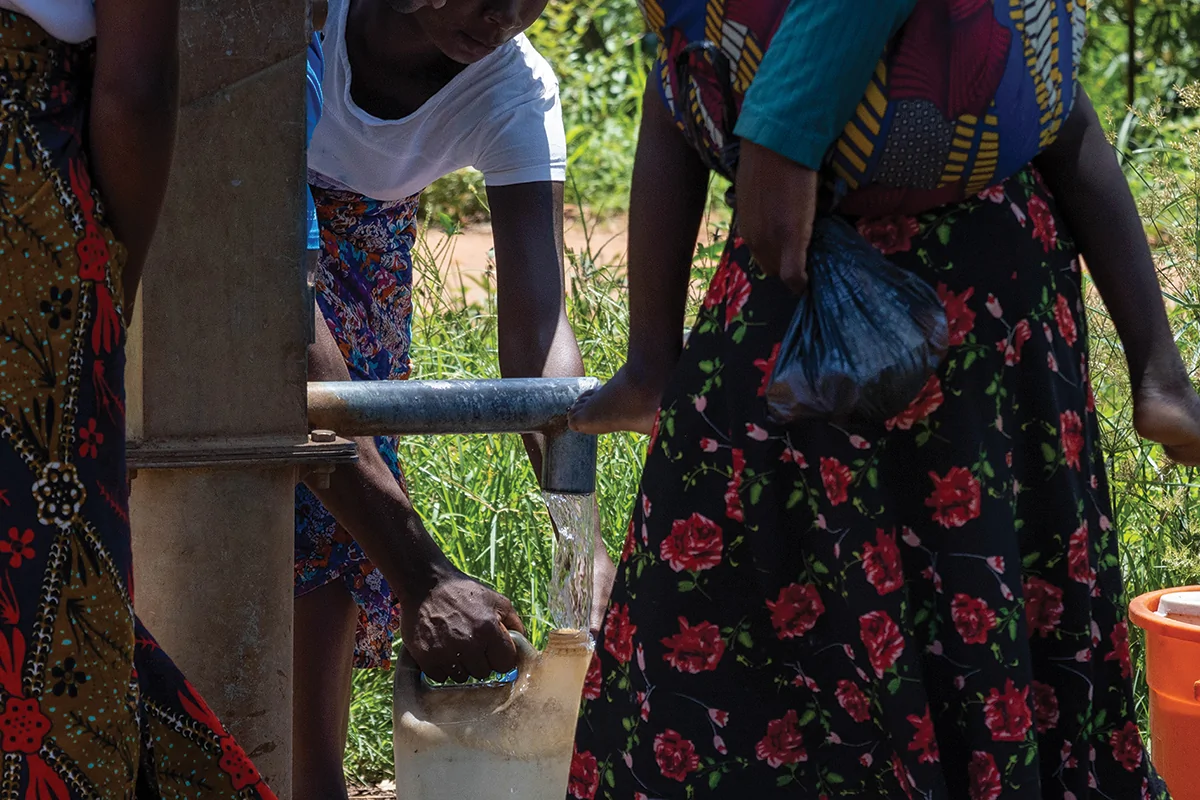
Challenges of collecting water from the shared community borehole for use at the health facility. The image illustrates the process of obtaining water from an external community water source. As the borehole is shared among many users (four can be seen in this photograph) and is not dedicated to the health facility, patients and guardians must often queue, wait their turn, and repeatedly carry heavy containers of water back to the facility, some holding up to 20 litres.

Guardians also faced a heavy burden, having to balance patient care with fetching water (Figure 2). Stakeholders explained that boreholes were often crowded, leading to long wait times and added stress. While the borehole at Thyolo DH was reported as reliable, filling a jerry can or bucket could take up to two hours due to the queue, and multiple trips were required daily. At Chimvu HC, guardians had to travel to an off-site community borehole, with roundtrips – including queuing and water collection – sometimes taking up to an hour. Most guardians made at least three trips a day. Stakeholders further explained that when staff were too busy and guardians were unavailable, the expectant mothers themselves had to fetch water from outside boreholes, putting their health at risk.

#### Reduced efficiency and quality of care

These issues were reported to directly impact care delivery, reducing time with patients, delaying services and creating patient backlogs.

Interviewees also acknowledged difficulties in maintaining infection prevention and control (IPC) practices due to a lack of water. During emergencies at the hospital or when patient numbers were high (e.g. up to three deliveries at once), staff stated they had to compromise on cleaning standards (both instruments and the environment). One interviewee noted that managing multiple patients at the same time made it nearly impossible for staff to wash their hands regularly using a bucket in a rushed setting.

Patients reported being unable to wash themselves after giving birth. A patient at Chimvu HC, who delivered at night, shared that she had to keep her soiled clothes on until the next morning, as she had no access to water until her guardian brought some. Another reported issue by stakeholders and interviewees was the potential for contamination from patients using the same buckets of water for multiple purposes, such as cleaning and drinking. For staff, the challenge was the lack of adequate storage facilities for water collected from the borehole.

These issues heightened the risk of infections for mothers. Staff at Thyolo DH noted that urinary tract infections (UTIs) were common in the antenatal ward, sepsis cases were reported in several wards, and there were frequent gaping wounds and post-op infections as IPC procedures were not consistently followed due to lack of water. Similarly, staff at Chimvu HC noted that women often developed complications after giving birth at the facility, but these were difficult to track consistently because they typically emerged after discharge.

#### Ripple effects

Beyond affecting care delivery, stakeholders explained that the lack of water also led to other consequences for patients. For example, some expectant mothers preferred using latrines outside rather than the toilets inside the health facilities due to their poor cleanliness, exposing them to additional risks. There were cases where women, in advanced labour, delivered their babies in the latrines after going outside to relieve themselves. These women had to give birth unassisted and in unsanitary conditions. Healthcare workers also reported that some patients skipped or delayed taking their anti-malaria tablets – which in Malawi are used both for treatment of symptoms and for routine prophylaxis in pregnancy as mosquito nets are not always available – because they did not have drinking water. Some waited until they returned home to take them, while others discarded them. This contributed to poor outcomes, as women were at risk of contracting malaria either during their stay at the health facility or on the way home after discharge. There were also reports of patients choosing to self-discharging early, despite guidelines, due to their basic water needs not being met and discomfort.

Stakeholders noted that these factors negatively impacted patient-provider relationships, including with guardians, who were often frustrated by the demands of fetching water for the health centres. Community health committees had tried to mediate and encourage guardians to assist but found it challenging.

### Wider contextual factors

Stakeholders also identified the range of factors that could influence the introduction of the technology, in line with the PESTLE approach [31], encompassing political, economic, social, technological, legal and environmental aspects, as summarised in Table 3 below.

**Table 3.** Contextual factors.

Factors	Risks	Mitigation
Political	Political interference by commercial interests, including those controlling water distribution to district hospitals, could hinder the introduction of alternative water provision solutions in the region.	Best to prioritise sites not connected to the main piped water supply at this initial stage.
Economic	Costs associated with the procurement of essential materials for manufacturing of the technology.	Important to prioritise use of locally available labour, materials and supply chains.
Social	Poor culture of rainwater harvesting, misconceptions over rainwater and lack of awareness of potential benefits of treated rainwater.	Introduction of the technology without community engagement is ill-advised as there may be resistance and suspicion toward ‘outsiders’. Sensitisation and education can help.
Technical	Variations in annual rainfall and sunshine patterns, which could affect the technology’s efficiency or consistency in performance.	Necessary to monitor variations in performance to track influence of weather changes.
Legal	Potential legal challenges arising from the introduction of alternative water solutions.	Need to liaise with relevant government agencies and sectoral players in this regard once the prototype is finalised.
Environmental	The potential environmental risks of flooding in the region, as seen during Cyclone Freddie in March 2023, which could damage infrastructure or affect water treatment processes.	While this risk cannot be entirely avoided, choice of materials and constructions could help, e.g. use plastic rather than concrete tanks.

### Ideation and intervention design

Based on the eight defined suitability criteria (covering water access, needs, and technical specifications), Chimvu Health Centre and Thekerani Rural Hospital were chosen as the two pilot sites to test the technology. The co-design was an iterative and continuous process of engagement. It began with the initial situation assessment reported above, followed by the presentation of preliminary findings to stakeholders, incorporation of their feedback, identification of remaining gaps, and joint refinement through repeated site visits and ongoing discussions with healthcare staff and other stakeholders. Insights generated through these interactions were progressively integrated into the final intervention. This collaborative process provided an opportunity to identify preferred features for the proposed technology while ensuring that its introduction at the study sites aligned with local cultural norms, end-users’ preferences and existing healthcare practices. The following needs and priorities were agreed upon:

- Ensure direct access to the treated water within the maternity unit of the facility.
- Prioritise the most critical area within the maternity unit: the delivery room.
- Maximise the technology’s capacity to harvest large quantities of rainwater, as healthcare facilities reported higher-than-expected unmet needs.
- Implement targeted measures to address potential behavioural barriers, including mistrust of new technologies and reluctance to use rainwater.

We combined feedback from end-users (healthcare providers and patients) and stakeholders with insights from behavioural science to create an implementation plan. Design thinking informed the development of the technology features and its deployment plan. The modifications made are summarised in Table 4.

**Table 4.** Changes to the technology design and deployment strategy based on the Co-creation process.

Original proposal/ technology specifications suggested	Modifications made based on co-creation and stakeholder feedback
Focus on larger facilities (e.g. district hospitals)	Focus shifted to health centres without piped water supply due to (i) the greatest gaps in water access, and (ii) to minimise potential interference from commercial interests
Target: maternity unit	Target refined to focus initially on the delivery room, identified as the area of most urgent need; with potential later expansion to the wider maternity unit (wards) and beyond
Clean water accessed directly from the SODIS system located just outside the maternity unit – from storage tank with bottom outlet	Addition of pipework connecting the SODIS system (clean water tank) directly to the delivery room sink, enabling faster access to water and reducing the need for manual transport from outside. This would also reduce the burden on patients’ guardians to fetch water for the delivery room (on top of water for their own needs)
Provision of one rainwater harvesting tank (10,000 litres), subject to confirmation	Provision of two rainwater harvesting tanks (total capacity 20,000 litres), as reported water needs higher than expected
Construction work by main contractor	Involve community members in construction to promote stronger sense of ownership
Provision of comprehensive initial training on operation and maintenance of the SODIS system	Inclusion of refresher training on operation and maintenance of the SODIS system as needed, recognising staff rotation and workforce turnover may affect continuity
Presence of on-site security sufficient to protect the SODIS system	Add fencing to the SODIS system for extra security
Behaviour change messaging focused primarily on promoting technology uptake	Behaviour change messaging expanded to address mistrust of new technologies and misconceptions regarding rainwater use
Focus mostly on healthcare workers	While healthcare workers remain the primary audience, messaging was broadened to include guardians and the wider community due to their role in supporting external water collection and their influence on attitudes toward rainwater

This plan was then enhanced with a range of Behaviour Change Techniques (BCTs) [15,32] to influence the primary behavioural objective: to promote the use of the treated rainwater produced by the SODIS system among healthcare workers in the labour ward, particularly for handwashing and other infection prevention and control practices. The behaviour change component was therefore designed to support the adoption and consistent use of this water source as part of routine clinical care. Since acceptance of the technology could be influenced by broader cultural beliefs, this also required action (see Table 4).

**Figure 3.**
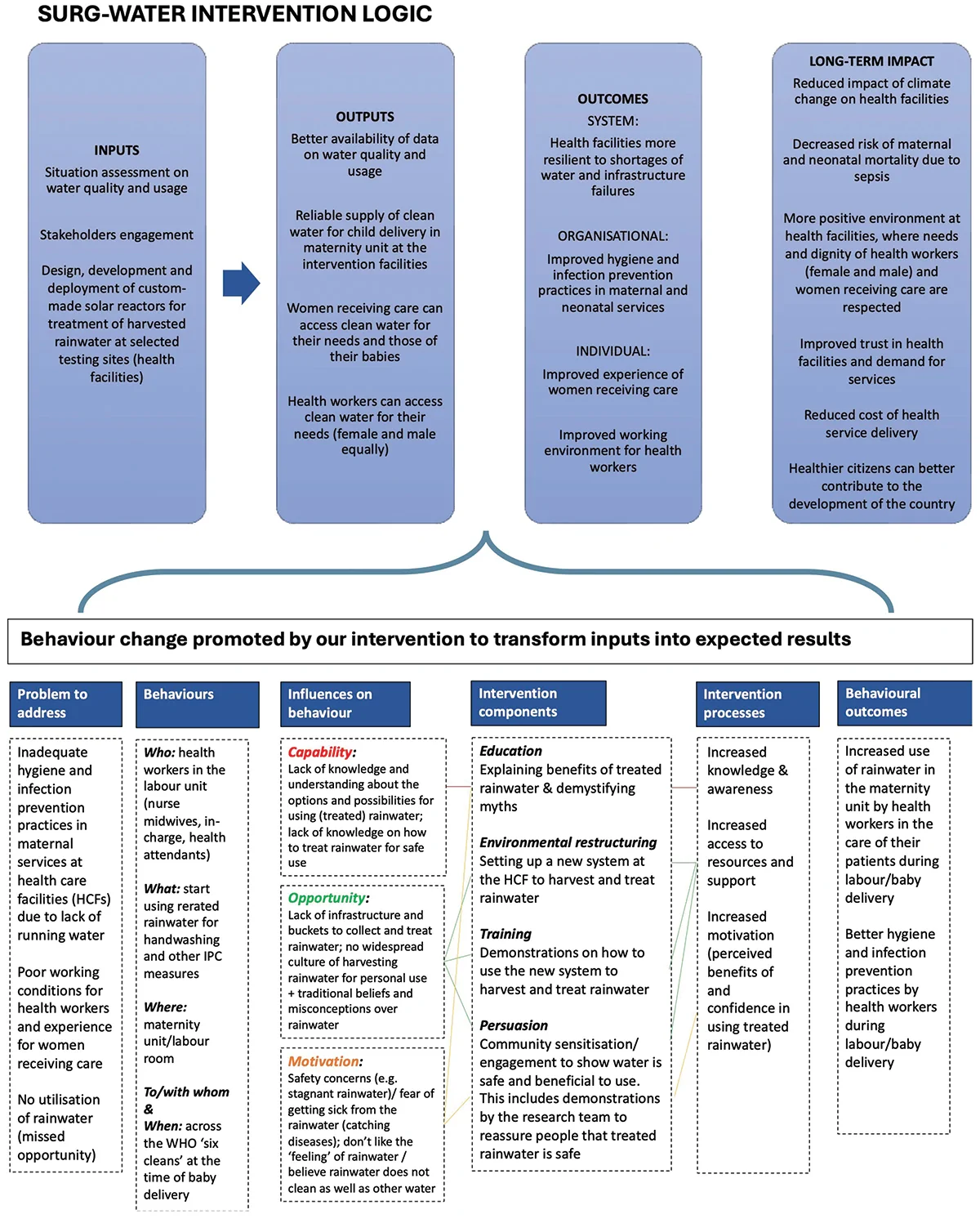
Intervention logic.

As a result, the final intervention package encompassed several key elements (see Figure 3):

- A flexible and user-friendly water treatment system, designed to be easily adapted to varying local infrastructure and environmental conditions, while ensuring the capacity to meet identified needs (e.g. large rainwater harvesting tank size) and minimal maintenance.
- Addressing behavioural barriers, such as mistrust of new technologies and resistance to change, through targeted interventions like community education and peer advocacy.
- A local capacity-building plan, including customised training, enabling technicians, healthcare providers, and community members to independently operate and maintain the system.
- Sensitisation of local communities, leveraging leaders to address concerns, promote the system’s benefits, and encourage collective participation.

The intervention logic is illustrated in Figure 3.

## Discussion and conclusion

Consistent with international reports [1], our study confirms that access to water continues to be a significant problem for HCFs in settings like Malawi. Our previous research has shown that this issue is widespread across the country, with 70% of district hospitals lacking reliable access to running water, and similarly affects neighbouring countries such as Zambia and Tanzania [25]. However, this study adds in-depth evidence of the challenges faced across frontline services – district hospitals and primary care health facilities- and implications for staff, patients, and service delivery, uncovering previously undocumented aspects of the issue. Along with the direct effect of water scarcity on health outcomes, a key finding is the reliance on guardians fetching water as a coping strategy by some healthcare facilities. This practice places an additional burden on patient guardians as well as strains provider–patient relationships, delays service delivery and reduces time with patients. This highlights that water scarcity at HCFs is a complex issue, with multi-layered ramifications, that demands equally complex solutions. By unpacking these dynamics, our study informs both technology design and implementation strategies that are grounded in real-world health system constraints.

We agree with other authors that such solutions should be built on intentional community participation [33]. This is important not only to align with local needs and priorities [34], but also to understand and acknowledge the power dynamics, socio-cultural and other factors that govern relations across actors and institutions in the water, and WASH, system [12,34]. Our experience reinforces the value of engaging users not just as informants, but as co-creators, which supports more legitimate and actionable outcomes.

From a methodological perspective, our experience underscored the value of combining design thinking with behavioural science to develop a user-centred and contextually relevant solution. The iterative, participatory design process in our case was crucial in ensuring that the technology was developed with direct input from end-users and stakeholders. This aligns with translational research principles by embedding ‘translation planning’ and ‘user-researcher conversations’ from the outset, rather than as downstream activities. This approach allowed for continual refinement of the design based on real-world insights, ensuring the solution addressed the unique challenges and constraints faced by healthcare providers and patients in rural Malawi. The final product is a simple technology, utilising freely available natural resources such as solar UV, gravity and rainwater. Based on the findings from this study, its technical features have been adapted to maximise ease of use. Importantly, its deployment strategy has evolved into a multi-component intervention that includes training, educational engagement, and community sensitisation, highlighting a broader pathway to implementation. This would not have been achievable without this foundational work, which goes beyond conventional research approaches and is a novelty in the SODIS research.

Indeed, studies from other countries [35,36] have highlighted that while solar disinfection may be widely accepted by target communities, simply providing physical resources and training is insufficient to ensure the sustained use of the technology. For instance, a study on solar disinfection using polyethylene terephthalate (PET) bottles in Ethiopian households found that close follow-up by local field workers – who lived in the same villages as the participants – considerably improved compliance compared to other settings, even though this support was primarily intended to ensure proper use of the bottles, rather than to directly motivate participants [37]. Similarly, a study in Nepal noted that follow-up visits, though intended solely for data collection, had a positive effect on participant engagement [35]. In an evaluation of an SODIS intervention in South Africa, Du Preez et al. found that a statistically significant reduction in dysentery was achieved only in households with higher motivation [36]. These studies point to the critical role of motivational and educational elements in the success of interventions, even though these aspects were not explicitly prioritised in the designs.

Studies that specifically targeted these elements yielded more promising results. For example, Mosler et al. [38] in Zimbabwe evaluated various approaches to promoting SODIS and found that combining household promotion with persuasive strategies, followed by interventions focused on maintaining new behaviours, led to sustained use of the technology over time.

However, none of these studies has examined this topic within healthcare settings, which, as mentioned earlier, are inherently more complex than individual household contexts due to factors such as the involvement of multiple stakeholders, institutional challenges and constraints, and the need to consider how practices and behaviours impact patient health outcomes. By applying the behavioural insights, our study contributes to closing a critical translational gap in water-related health interventions.

In our experience, effectively integrating these human and behavioural elements requires incorporating them from the onset of the research, ensuring a purposeful and methodologically sound approach throughout the intervention. Moreover, rural communities like the ones in this case study are particularly vulnerable, making it essential for researchers to manage relationships and expectations carefully for the intervention to work [39].

In conclusion, our experience highlights the critical importance of adopting a holistic, human-centred approach when tackling complex issues like water scarcity in healthcare settings. By integrating technical solutions with a deep understanding of local needs, behaviours and power dynamics, we can not only improve healthcare delivery but also build trust and foster long-term, sustainable change in rural communities. Our model offers a methodological template for translating research into action, combining co-design, behavioural insights, and adaptive learning to build real-world relevance. Ultimately, the lessons learned here emphasise the need for thoughtful, context-specific approaches to intervention design to maximise the chances of lasting impact. Despite the limitations of a small sample size and the inability to generalise the results due to the nature of the research, this case study provides methodological insights that can inform and guide the design of future interventions in similar resource-constrained settings, where insufficient or wrong investments can have profound consequences. Hence, a clear pathway from problem identification to solution design, such as the one here illustrated, can help practitioners and decision-makers make more informed and impactful decisions.

## Supplementary Material

Supplementary mateterial 2_STROBE.doc

Supplementary_Material_1_CLEAN.docx

## Data Availability

Data for this research are included in this paper (and in its Supplementary Material).
