# Supplementary material for: Integrating behavioural science and design thinking to develop a water treatment innovation in Malawi healthcare: a case study of the intervention design process
Source: Glob Health Action. 2026 Sep 9;19(1):2726126. doi: 10.1080/16549716.2026.2726126 (PMC13560407; doi:10.1080/16549716.2026.2726126)
Supplement: Supplementary file 1 [file ZGHA_A_2726126_SM1129.docx]

# Supplementary Material 1

Table S1. Results of the Site Inspections

| **Site inspection checklist and scores - range 0 (lease suitable - 5 most suitable)** | | | | | | |
| --- | --- | --- | --- | --- | --- | --- |
| **Criteria** | **Mulanje district hospital** | **Thyolo district hospital** | **Chickwawa district hospital** | **Bvumbwe health centre** | **Chimvu health centre** | **Thekerani rural hospital** |
| Water access problems | 1 | 3 | 1 | 1 | 5 | 5 |
| Suitable roof for rainwater collection | 2 | 5 | 1 | 2 | 5 | 5 |
| Available sunlight  (lack of shade) | 3 | 5 | 2 | 3 | 5 | 5 |
| East-West orientation | 2 | 5 | 1 | 2 | 5 | 5 |
| 24/7 on-site security | 2 | 4 | 1 | 2 | 3 | 4 |
| Available space for  SURG-Water tank and reactor | 2 | 5 | 2 | 3 | 5 | 5 |
| Ease of access (e.g. during the rainy season and considering road conditions) | 4 | 5 | 3 | 5 | 1 | 5 |
| Possible impact (i.e. severity of water challenges vs. demand for maternal health services) | 2 | 3 | 3 | 2 | 5 | 5 |
| **Total** | **18** | **35** | **14** | **20** | **34** | **39** |
